# Nutritional support and brain tissue glucose metabolism in poor-grade SAH: a retrospective observational study

**DOI:** 10.1186/cc11160

**Published:** 2012-01-25

**Authors:** J Michael Schmidt, Jan Claassen, Sang-Bae Ko, Hector Lantigua, Mary Presciutti, Kiwon Lee, E Sander Connolly, Stephan A Mayer, David S Seres, Neeraj Badjatia

**Affiliations:** 1 Columbia University, Department of Neurology, Milstein Hospital, 177 Fort Washington, Suite 8-300, New York, NY 10032, USA; 2 Columbia University, Department of Neurological Surgery, Neurological Institute, 4th Floor, 710 West 168th Street, New York, NY 10032, USA; 3 Columbia University, Department of Medicine, Physicians and Surgeons, Box 85 - Preventive Med, Mail Code: 9, New York, NY 10032, USA; 4 New York Presbyterian Hospital, Department of Nursing, Milstein Hospital, 177 Fort Washington, Neuro-ICU, New York, NY 10032, USA

## Abstract

**Introduction:**

We sought to determine the effect of nutritional support and insulin infusion therapy on serum and brain glucose levels and cerebral metabolic crisis after aneurysmal subarachnoid hemorrhage (SAH).

**Methods:**

We used a retrospective observational cohort study of 50 mechanically ventilated poor-grade (Hunt-Hess 4 or 5) aneurysmal SAH patients who underwent brain microdialysis monitoring for an average of 109 hours. Enteral nutrition was started within 72 hours of admission whenever feasible. Intensive insulin therapy was used to maintain serum glucose levels between 5.5 and 7.8 mmol/l. Serum glucose, insulin and caloric intake from enteral tube feeds, dextrose and propofol were recorded hourly. Cerebral metabolic distress was defined as a lactate to pyruvate ratio (LPR) > 40. Time-series data were analyzed using a general linear model extended by generalized estimation equations (GEE).

**Results:**

Daily mean caloric intake received was 13.8 ± 6.9 cal/kg and mean serum glucose was 7.9 ± 1 mmol/l. A total of 32% of hourly recordings indicated a state of metabolic distress and < 1% indicated a state of critical brain hypoglycemia (< 0.2 mmol/l). Calories received from enteral tube feeds were associated with higher serum glucose concentrations (Wald = 6.07, *P *= 0.048), more insulin administered (Wald = 108, *P *< 0.001), higher body mass index (Wald = 213.47, *P *< 0.001), and lower body temperature (Wald = 4.1, *P *= 0.043). Enteral feeding (Wald = 1.743, *P *= 0.418) was not related to brain glucose concentrations after accounting for serum glucose concentrations (Wald = 67.41, *P *< 0.001). In the presence of metabolic distress, increased insulin administration was associated with a relative reduction of interstitial brain glucose concentrations (Wald = 8.26, *P *= 0.017), independent of serum glucose levels.

**Conclusions:**

In the presence of metabolic distress, insulin administration is associated with reductions in brain glucose concentration that are independent of serum glucose levels. Further study is needed to understand how nutritional support and insulin administration can be optimized to minimize secondary injury after subarachnoid hemorrhage.

## Introduction

Hyperglycemia, defined as serum glucose > 11 mmol/l after subarachnoid hemorrhage, is a common finding and is an independent predictor of long term poor outcome [1,2]. It is believed that the surge in serum glucose immediately after injury is related to a complex interrelationship between sympathetic and inflammatory mediators, acquired insulin resistance and administration of dexamethasone [3].

While such observational data argue for tight glycemic control, recent studies investigating the relationship between serum and brain glucose levels have found that intensive insulin protocols targeted to a tight serum glucose level (4.4 to 6.1 mmol/l) may compromise cerebral metabolism by leading to concomitant critical reductions in interstitial brain glucose levels [4,5].

Each of these studies did not take into account the impact that nutritional support may have upon outcomes related to tight glycemic control. The adequate delivery of non-protein calories was rigorously maintained in randomized controlled trials of surgical and medical ICU patients [6-8], and a recent single center study of SAH patients found that a bolus delivery of enteral nutrition significantly impacted brain glucose levels [9].

We sought to understand the impact enteral nutrition has upon the relationship between serum and brain interstitial glucose levels in a cohort of subarachnoid hemorrhage patients treated with institutional protocol targeting serum glucose levels between 5.5 to 7.8 mmol/l. We hypothesized that higher non-protein caloric delivery would result in higher interstitial brain glucose levels and reduce the incidence of brain metabolic crisis.

## Materials and methods

### Patient selection and data collection

Between June 2006 and August 2010 data were collected from 50 consecutive aneurysmal subarachnoid hemorrhage (SAH) patients admitted to the neurological ICU at Columbia University Medical Center, who underwent cerebral metabolism monitoring as part of their clinical care. All patients were enrolled in the Columbia University SAH Outcomes Project. This observational study was approved by the Columbia University Medical Center Institutional Review Board, and in all cases written informed consent was obtained from the patient or a surrogate. Details regarding data collection have been described previously [10]. The diagnosis of SAH was established on the basis of an admission CT scan or by presence of xanthochromia in the cerebrospinal fluid. Patients with secondary SAH related to trauma, rupture of an cerebral arteriovenous malformation (AVM), or other causes; age < 18 years; or admission > 14 days after SAH onset were excluded from the study.

### Clinical and radiographic variables

The calendar day of the index bleeding event was designated SAH Day 0. We recorded baseline demographic data, location and size of the aneurysm, angiographic and transcranial Doppler ultrasonography (TCD) findings, and mode of aneurysm treatment (clipping versus coiling). Neurological status on admission was assessed with the Hunt-Hess scale [11], and the Glasgow Coma Scale (GCS; [12]). We also assessed the Acute Physiology and Chronic Health Evaluation II (APACHE II) scale [13] and calculated a physiological sub-score by subtracting the GCS score, age and chronic health elements from the total score [14]. Admission and follow-up CT scans were independently evaluated by a study neurointensivist for the amount and location of subarachnoid blood (SAH sum score, scaled 0 = no blood, 30 = all cisterns and fissures completely filled) [15], intraventricular blood (IVH sum score, scaled 0 = no blood, 12 = all ventricles completely filled) [16], the presence and degree of hydrocephalus [17], the presence of cerebral edema [18], and cerebral infarction.

### Clinical management

Clinical management conformed to guidelines set forth by the American Heart Association [19]. External ventricular drainage (EVD) was placed in all patients with symptomatic hydrocephalus or intraventricular hemorrhage with a reduced level of consciousness. All patients were followed with daily or every-other-day TCD, received oral nimodipine and intravenous hydration with 0.9% saline and 250 ml of 5% albumin solution to maintain a central venous pressure > 5 cm H_2_O. Clinical deterioration from delayed cerebral ischemia (DCI) was treated with hypertensive hypervolemic therapy (HHT) to maintain systolic blood pressure (SBP) between 160 and 220 mm Hg, as required to reverse the neurological deficit. Comatose patients were mechanically ventilated and treated with propofol, fentanyl or dexmedetomidine to facilitate ventilator synchrony, with interruptions in sedation for clinical assessments at least twice daily. Computed tomography (CT) was performed serially when clinically needed. All patients with clinical deterioration underwent CT or magnetic resonance imaging (MRI) scanning to identify causes of deterioration other than vasospasm whenever clinically feasible. When clinical evidence of DCI persisted for more than two hours despite HHT, cerebral angiography was used to identify vasospasm and balloon angioplasty or intra-arterial administration of verapamil was performed whenever feasible.

### Nutritional management

Nutrition support was begun within 24 hours after aneurysmal repair. Enteral nutrition was the preferred route and was provided via a naso-duodenal tube starting within the first 24 hours of admission. All nutritional formulas were started at 50% of goal and were titrated by 25% every four hours until targeted caloric intake was achieved. Feeds were held for two hours if 100 cc of residual feeds were noted in the FARRELL^® ^valve bag (Corpak MedSystems, Inc., Buffalo Grove, IL, USA), and resumed once the FARRELL^® ^emptied. The target goal was 25 calories/kg/day of ideal body weight. No parenteral nutrition was given [20]. The type of enteral formula used was determined by the clinical nutritionist. All nutrition data were adjusted for actual body weight.

### Serum glucose management

Serum glucose was measured with the Sure Step Flexx system (Lifescan, Milpitas, CA, USA) and the target range was between 5.5 and 7.8 mmol/l (100 to 140 mg/dL) as part of an institutionally approved tight serum glucose control protocol using intravenous insulin infusion (Humulin, Novo Nordisk, Princeton, NJ, USA) [7].

### Cerebral metabolism

A CMA 70 microdialysis catheter with 10 mm membrane length (CMA Microdialysis^®^, Stockholm, Sweden) was inserted at the bedside through the same triple-lumen bolt as intracranial pressure (ICP) and brain tissue oxygen tension (P_bt_O_2_). Immediately after the procedure, a brain CT scan was performed in each patient to confirm the proper location of the microdialysis catheter in the normal appearing white matter. The CMA 106 microdialysis perfusion pump (CMA Microdialysis^®^) was used to perfuse the interior of the catheter with sterile artificial cerebrospinal fluid (Na^+ ^148 mmol/L, Ca^2+ ^1.2 mmol/L, Mg^2+ ^0.9 mmol/L, K^+ ^2.7 mmol/L, Cl^- ^155 mmol/L) at a rate of 0.3 μl/minute. Samples were collected in microvials approximately every 60 minutes and immediately analyzed at the bedside for glucose, lactate and pyruvate with the CMA 600 analyzer (CMA Microdialysis^®^). At least one hour passed after the insertion of the probe and the start of the sampling to allow for normalization of changes due to probe insertion. The analyzer was automatically calibrated on initiation and every six hours using standard calibration solutions from the manufacturer. Quality controls at three different concentrations for each marker were performed daily.

### Statistical analysis

Caloric intake was quantified for enteral nutrition, dextrose infusions and propofol infusions. The volume (mL) of enteral nutrition delivered each hour was recorded and multiplied by the appropriate factor provided for each specific formulation. Hourly volumes (mL) of dextrose were converted into grams of dextrose and multiplied by 3.4 calories/gram dextrose. Propofol volumes (mL) were converted to calories by a factor of 1.1 calories/mL. Metabolic crisis was defined as a lactate/pyruvate ratio greater than 40 [5]. Comparisons of pooled data were carried out using a generalized linear model (GLM) using a normal distribution and identity link function and were extended by generalized estimating equations (GEE) using the autoregressive process (AR-1) [21] to handle repeated observations within subject. Data were transformed to meet assumptions of normality or converted into ordinal factors based on median or quartile values. Brain glucose was transformed using a square root transformation (bGlu_sqrt) whereas a Log transformation was applied to the lactate/pyruvate ratio (LPR_log). A Cook's distance was calculated in a linear analysis of bGlu_sqrt and LPR_log to identify outliers and a filter was applied using a cutoff of greater than 4/N [22] to remove the remaining data points that would distort linear analysis. SPSS 18 software^® ^(IBM SPSS Inc., Chicago, IL, USA) was used for data analysis. A *P-*value < 0.05 was considered statistically significant.

## Results

### Baseline characteristics and nutrition

Fifty aneurysmal SAH patients met study criteria and were monitored for a total of 5,445 hours (108 +/- 73 hours/patient) between June 2006 and August 2010. The median Hunt &Hess Score on admission was 4 (IQR: 3, 5) and mean APACHE II score was 22 +/- 8 (Table 1). Neuromonitoring began within four days after SAH in all but four patients (median = 2 days, IQR = 1 to 3.5 days). Enteral nutrition started on SAH bleed Day 4 +/- 2. The daily mean non-protein caloric intake was 14.8 ± 10.7 cal/kg and daily nitrogen intake was 0.087 ± 0.085 g/kg. The daily mean caloric intake from enteral tube feeds during the study period was 10.63 +/- 6.9 calories/kg/day, from dextrose 0.85 +/- 1.4 calories/kg/day, and from propofol 2.27 +/- 2.26 calories/kg/day totaling overall 13.77 +/- 6.95 calories/kg/day (Table 2). Patients had a mean serum glucose of 7.9 +/- 1 mmol/l, brain glucose of 1.21 +/- 0.60 mmol/l, and LPR of 37 +/- 14. Higher insulin administration (IU) was associated with higher caloric intake from enteral tube feeds (Wald = 9.49, *P *= 0.009).

**Table 1:** Baseline characteristics of subarachnoid hemorrhage patients (*N *= 50)

Characteristic	**Value**^**1**^
Age, years	51.2 (14.7)
Women	32 (64%)
Ethnicity	
White, non-Hispanic	18 (36%)
White, Hispanic	19 (38%)
Black	8 (16%)
Asian	5 (10%)
Past medical history	
Diabetes mellitus	5 (10%)
Hypertension	17 (34%)
Liver Disease	2 (4%)
Kidney Disease	0 (0%)
Body Mass Index, *kg/m^2^*	26 (5)
APACHE II score^2^	22 (8)
Glasgow Coma Scale	6 (5)
Aneurysm size, mm^3^	7.7 (3.8)
Aneurysm clipping	26 (52%)
Admission Hunt Hess Grade	
2	3 (6%)
3	7 (14%)
4	15 (30%)
5	25 (50%)
Modified Fisher Score	
0	1 (2%)
1	4 (8%)
2	9 (18%)
3	14 (28%)
4	22 (44%)

^1 ^All continuous measures are shown as mean (SD), ordinal measures as median (IQR), and nominal measures as N(%). ^2 ^Acute Physiological and Chronic Health Evaluation Score. ^3 ^One patient had a dysplastic aneurysm that was not measured for diameter.

**Table 2:** Nutrition

Characteristic	Mean (SD)
Enteral Nutrition	Calories/Kg/Hour
Carbohydrates	0.224 (0.142)
Protein	0.085 (0.056)
Fat	0.133 (0.099)
Intravenous Infusions	
Dextrose	0.035 (0.057)
Propofol	0.095 (0.094)

### Serum glucose

**Figure 1:**
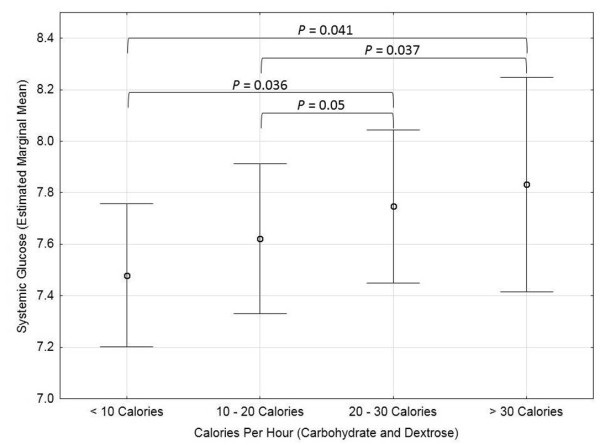
**Calories and Systemic Glucose**. Range plot of positive relationship between serum glucose to caloric intake from carbohydrates and dextrose (Wald = 27.631, *P *< 0.001). Serum glucose estimated marginal mean and 95% confidence interval is presented.

**Figure 2:**
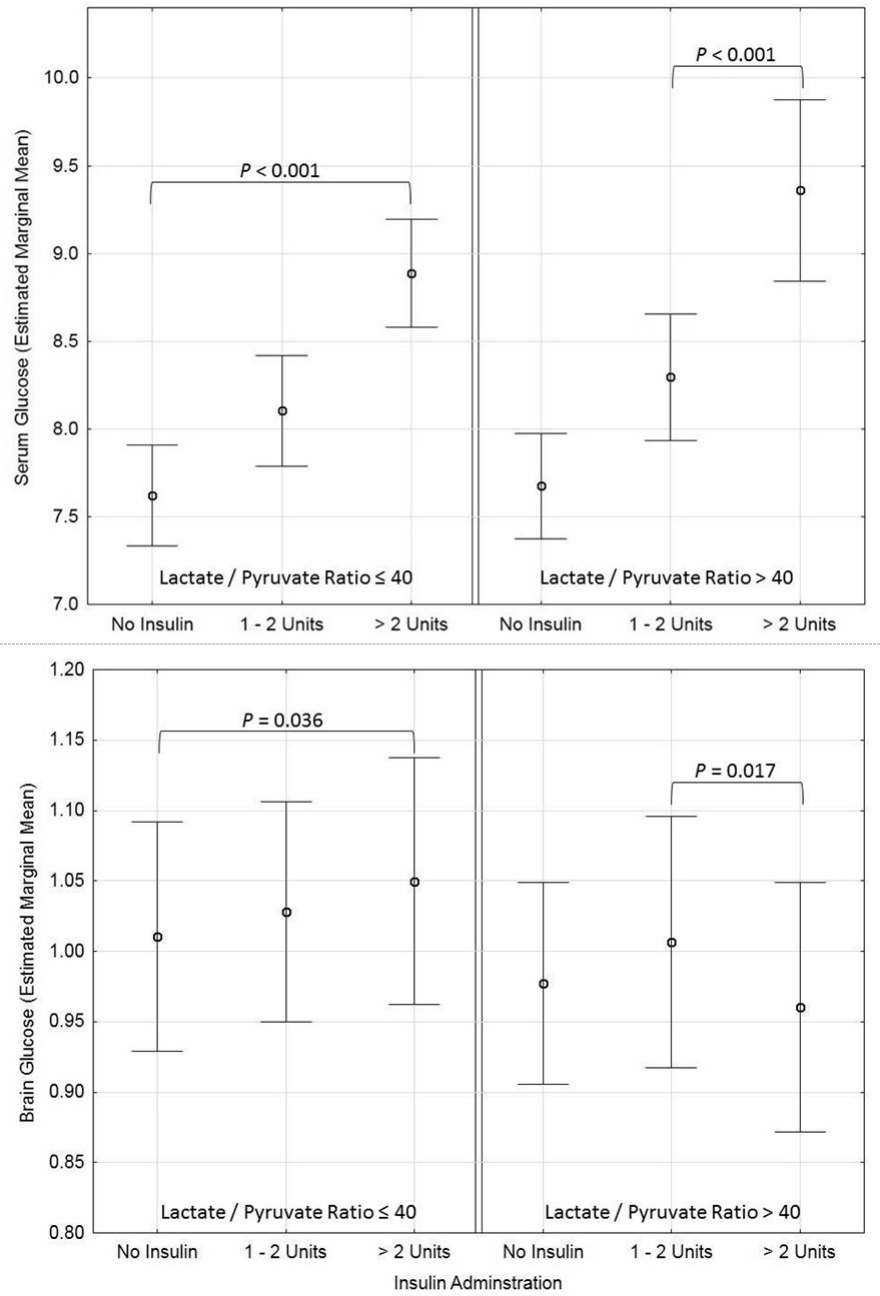
**Calories and Brain Glucose**. Observed association between insulin and brain glucose is modified by presence of metabolic crisis at the probe location. Brain glucose estimated marginal mean and 95% confidence interval is presented.

On univariate analysis, higher serum glucose levels were associated with higher brain glucose concentrations (Wald = 30.4, *P *< 0.001), doses of insulin administration (IU/hr: Wald = 92.25, *P *< 0.001), body mass index (Wald = 213.47, *P *< 0.001), and lower body temperature (Wald = 4.1, *P *= 0.043). Combined calories received from carbohydrates and dextrose administration were significantly related to serum glucose concentrations (Wald = 27.631, *P *< 0.001, Figure 1) but associations were not found for total calories from enteral feeding (Wald = 2.173, *P *= 0.337), dextrose administration (Wald = 1.434, *P *= 0.488), or propofol (Wald = 5.284, *P *= 0.071). Multifactorial analysis revealed that increased calories from enteral feeding (Wald = 6.07, *P *= 0.048), higher insulin doses (Wald = 108.2, *P *< 0.001), and presence of brain metabolic crisis (LPR > 40) (Wald = 4.4, *P *= 0.036) were significantly associated with higher serum glucose concentrations. Insulin administration modified the relationship between serum glucose and enteral calories (Wald = 9.96, *P *= 0.041) such that serum glucose concentrations increases were smaller in magnitude in the presence of insulin administration, but insulin did not impact the relationship between serum glucose and brain metabolic crisis (Wald = 3.48, *P *= 0.176) (Table 3, Figure 2).

**Table 3:** Serum glucose: multifactorial associations

Characteristic	Estimate (95% Confidence Interval)	*P-*Value
Enteral feeds (cal/kg/hour)		
0 to 0.068	(Reference)	
0.069 to 0.697	0.115 (-0.065, 0.296)	0.21
0.70 to 2.16	0.249(-0.021, 0.520)	0.07
Insulin administration (units)		
0	(Reference)	
1 to 2	0.678 (0.412, 0.944)	< 0.001
> 2	1.526 (1.124, 1.927)	< 0.001
Lactate/pyruvate ratio ≤ 40	-0.472 (-0.912, -0.032)	0.036
Enteral feeds * Insulin administration		
0 to 0.068 Cal/kg/hour * 0 units	(Reference)	
0.069 to 0.697 Cal/kg/hour * 1 to 2 units	-0.356 (-0.676, -0.036)	0.029
0.069 to 0.697 Cal/kg/hour * > 2 units	-0.562 (-0.980, -0.144)	0.008
0.70 to 2.16 Cal/kg/hour * 1 to 2 units	-0.232 (-0.646, 0.182)	0.272
0.70 to 2.16 Cal/kg/hour * > 2 units	-0.216 (-0.787, 0.355)	0.458
Insulin Administration * LPR ≤ 40		
Insulin Administration > 2 units * LPR ≤ 40	(Reference)	
Insulin Administration 1 to 2 units * LPR ≤ 40	0.160 (-0.035, 0.594)	0.436
Insulin Administration 0 units * LPR ≤ 40	0.419 (-0.067, 0.904)	0.091

### Brain glucose

Univariate analysis revealed that higher brain glucose was related to higher insulin doses (Wald = 24.68, *P *< 0.001) and body mass index (Wald = 97.98, *P *< 0.001), while lower brain glucose levels were associated with metabolic crisis (LPR > 40; Wald = 14.79, *P *< 0.001). There was no association observed between brain glucose levels and calories received from enteral feeding (Wald = 0.947, *P *= 0.623), propofol (Wald = 3.153, *P *= 0.207), dextrose (Wald = 5.76, *P *= 0.056) or body temperature (Wald = 3.05, *P *= 0.08). Multifactorial analysis revealed that enteral feeding (Wald = 1.743, *P *= 0.418) was not related to brain glucose concentrations after accounting for the positive relationship of serum glucose concentrations (Wald = 67.41, *P *< 0.001) and negative relationship of metabolic crisis (Wald = 4.61, *P *= 0.032) to brain glucose concentrations. In this model insulin administration (Wald = 3.15, *P *= 0.208) was not associated with brain glucose but was modified by the existence of metabolic crisis (Wald = 8.26, *P *= 0.016) such that in the presence of metabolic crisis, administration of greater than two units of insulin was associated with a relative reduction of brain glucose concentrations (Table 4, Figure 2).

**Table 4:** Brain glucose: multifactorial associations

Characteristic	Estimate (95% confidence interval)	*P-*Value
Enteral feeds (cal/kg/hour)		
0 to 0.068	(Reference)	
0.069 to 0.697	0.012 (-0.007, 0.032)	0.680
0.70 to 2.16	-0.006 (-0.036, 0.024)	0.213
Insulin administration (units)		
0	(Reference)	
1 to 2	0.018 (-0.014, 0.049)	0.275
> 2	0.039 (0.003, 0.076)	0.036
Serum glucose (mmol/L)	0.036 (0.027, 0.045)	0.000
Lactate/pyruvate ratio ≤ 40	0.089 (0.027, 0.152)	0.032
Insulin administration (units) * LPR ≤ 40		
Insulin administration > 2 units * LPR ≤ 40	(Reference)	
Insulin administration 1 to 2 units * LPR ≤ 40	-0.048 (-0.091, -0.005)	0.005
Insulin administration 0 units* LPR ≤ 40	-0.059 (-0.122, 0.004)	0.062

## Discussion

In this study of 50 poor-grade SAH patients, we found that our insulin protocol maintained serum glucose levels ≤ 7.8 mmol/l and similar to previous observations that low serum glucose levels were associated with brain metabolic crisis. We were unable; however, to directly demonstrate our central hypothesis that higher non- protein caloric delivery results in higher interstitial brain glucose levels. In contrast, Kinoshita *et al. *[9] demonstrated increases in serum and brain glucose concentrations two hours after a 250-calorie bolus of ENSURE^® ^(Abbott Laboratories, Inc., Columbus, Ohio, USA **{**). This discrepancy is likely due to a significantly lower caloric delivery in our patients (0.442 calories/kg/hour) in combination with our intravenous insulin protocol that is designed to maintain serum glucose levels within a range of 5.5 to 7.8 mmol/l. Kinosheta *et al. *provided bolus feeds that resulted in higher serum glucose levels (183.8 ± 26.2 mg/dL) that were actively controlled in our observational study. This is a limitation experimentally but does suggest that brain metabolism is not supported by enteral nutrition under normal clinical circumstances. Underfeeding is a common problem in critically ill patients and as we and others have shown, it is associated with higher rates of medical and infectious complications as well as poor long term outcome [23,24].

As expected, we observed greater administration of insulin as serum glucose levels increased. However, we were surprised to observe a different relationship between brain glucose levels and insulin doses. In 2011, Zetterling *et al. *[25] reported that insulin administration lowered cerebral microdialysis brain glucose and pyruvate concentrations, often to low levels, despite plasma glucose remaining above 6 mmol/L. While cerebral metabolism monitoring is used to detect acute events, in practice severity of the initial brain injury is the primary determinate of observed metabolic status [26]. An LPR greater than 40 has been established in the literature to represent a state of brain metabolic crisis (for example, [27]). We speculated that perhaps the effect of insulin on brain glucose concentration is altered by the severity of injury of the tissue being monitored. We found that when metabolic status was not critical (LPR < 40) that the brain glucose-insulin administration relationship was similar to the serum glucose-insulin administration relationship. In contrast, during metabolic crisis (LPR ≥ 40) a negative relationship was observed, such that higher insulin doses (> 2 IU/hr) were associated with a lower brain glucose level. While our observational study design restricts our ability to infer causal relationships between insulin and brain glucose, our observations do appear congruent with the findings of Zetterling *et al.*

It is physiologically plausible that the effect of insulin on brain glucose metabolism may be altered by brain injury. Previous experimental and clinical studies have shown that insulin increases glucose transport across the blood-brain barrier [28], promotes glucose storage as glycogen in astrocytes [29], and stimulates protein and RNA and DNA synthase in the brain [30,31]. High concentrations of endogenous insulin may enable brain cells to divert glucose metabolism to secondary pathways [32,33] that may be important to neuronal repair, including fatty acid and amino acid production, and the pentose phosphate shunt pathway, which may be important to protect against oxidative damage [34].

It is conceivable that protocol-driven clinical administration of insulin to maintain serum glucose concentrations may provide a dangerous false signal to injured brain tissue that excess glucose is available and can be diverted from ATP production to secondary pathways for tissue repair functions. Vespa *et al.*, [4] conducted a dual microdialysis positron emission tomography (PET) scan study in traumatic brain injury patients demonstrating that even though intensive insulin therapy resulted in lower brain glucose concentrations, the global metabolic rate of glucose did not change and that this corresponded with signs of energy failure, including oxygen extraction increases to near-ischemia level and brain glutamate and lactate pyruvate ratio increases. Two other studies found significant insulin-related reductions in brain glucose concentrations but did not find evidence that this resulted in increased metabolic distress [25,35]. However, in both studies the reported mean LPR was approximately 30. No study has looked at the relationship between brain glucose levels and insulin during cerebral metabolic distress (LPR > 40). Glucose metabolism of healthy brain tissue is stable and relatively unaffected by normal alterations of cerebral perfusion in contrast to that of injured brain tissue, which is very sensitive to such changes [36].

It is currently unknown if providing additional nutritional support to brain injured patients in combination with insulin therapy may be important to support increased cerebral energy demands and brain tissue recovery. The current cohort of patients, on average, received approximately half the amount of calories recommended by the American College of Chest Physicians' guidelines for critically ill patients (approximately 25 calories/kg/day) [20], but is consistent with reports of underfeeding of neurological critically ill patients elsewhere [37]. We speculate that the effect of insulin on brain glucose metabolism in the context of brain injury remains poorly understood and is in need of further study.

Our study has several limitations. The true impact of enteral nutrition on brain metabolism is difficult to determine due to the fact that serum glucose concentrations were actively controlled with insulin. Evaluating relatively small-to-moderate differences in calories received over time may also mask the effects of enteral nutrition on brain metabolism. Individual responses to increases in calories received via enteral nutrition and the impact of insulin infusions were not analyzed in this study. Although our findings are consistent with the few studies available in this area [4,9,25,35,38], a patient-specific analysis might lead to different conclusions. This study does not provide any data to clarify a mechanism for insulin-related reductions of brain glucose concentrations or suggest whether adequate caloric intake (for example, 25 calories/kg/day) might mitigate this effect. Prospective studies with controlled amounts of caloric delivery are required to adequately address these limitations.

## Conclusions

Our data suggest that receiving even modest, though typical [37], levels of nutritional support is an effective way to increase serum glucose concentrations in poor-grade SAH patients and, accordingly, results in increased insulin administration to maintain target serum glucose concentrations. Improved algorithms designed to avoid underfeeding will be necessary to fully understand the incidence, mechanism and impact of insulin-induced reductions in brain glucose concentrations.

## Key messages

• Higher caloric intake from enteral nutrition was associated with higher serum glucose concentrations and the amount of insulin administered.

• Caloric intake from enteral nutrition was not related to brain glucose concentrations after accounting for serum glucose concentrations.

• In the presence of metabolic distress, higher doses of insulin were associated with lower brain glucose levels.

• Further study is needed to understand how nutritional support and insulin administration can be optimized to minimize secondary injury after subarachnoid hemorrhage.

## Abbreviations

APACHE II: Acute Physiology and Chronic Health Evaluation-2; AR1: autoregressive process; AVM: cerebral arteriovenous malformation; bGlu_sqrt: square root transformation of brain glucose; CT: computed tomography; DCI: delayed cerebral ischemia; EVD: External ventricular drain; GCS: Glasgow Coma Scale; GEE: generalized estimation equations; GLM: general linear model; HHT: hypertensive hypervolemic therapy; ICP: intracranial pressure; IQR: interquartile range; IU: insulin administration; IVH: intraventricular hemorrhage; LPR: lactate to pyruvate ratio; LPR_log: log transformation of lactate-to-pyruvate ratio; MRI: magnetic resonance imaging; P_bt_O_2_: brain tissue oxygen tension; SAH: aneurysmal subarachnoid hemorrhage; SBP: systolic blood pressure; TCD: transcranial Doppler ultrasonography.

## Competing interests

The authors declare that they have no competing interests.

## Authors' contributions

JMS participated in the design of the study, directed data collection, performed the data analyses, led discussion of results interpretation and drafted the manuscript. JC participated in the collection of data, results interpretation and made critical revisions to the manuscript. SBK collected cerebral metabolism data and participated in drafting the manuscript. HL collected the clinical patient data and participated in results interpretation. MP collected ICU patient data and participated in results interpretation. KL, ESC and SAM participated in data collection, results interpretation and made critical revisions to the manuscript. DSS provided expertise in interpretation of nutrition results and made critical revisions to the manuscript. NB conceived of the study, participated in the collection of data, results interpretation, and drafting of the manuscript. All authors have read and approved the final manuscript.

## References

[B1] Badjatia N Topcuoglu MA Buonanno FS Smith EE Nogueira RG Rordorf GA Carter BS Ogilvy CS Singhal AB Relationship between hyperglycemia and symptomatic vasospasm after subarachnoid hemorrhage Crit Care Med 2005 33 1603 1609 quiz 1623 10.1097/01.CCM.0000168054.60538.2B 16003069

[B2] Frontera JA Fernandez A Claassen J Schmidt M Schumacher HC Wartenberg K Temes R Parra A Ostapkovich ND Mayer SA Hyperglycemia after SAH: predictors, associated complications, and impact on outcome Stroke 2006 37 199 203 16339481 10.1161/01.STR.0000194960.73883.0f

[B3] Rabinstein AA Hyperglycemia in critical illness: lessons from NICE-SUGAR Neurocrit Care 2009 11 131 132 10.1007/s12028-009-9240-x 19499351

[B4] Vespa P Boonyaputthikul R McArthur DL Miller C Etchepare M Bergsneider M Glenn T Martin N Hovda D Intensive insulin therapy reduces microdialysis glucose values without altering glucose utilization or improving the lactate/pyruvate ratio after traumatic brain injury Crit Care Med 2006 34 850 856 10.1097/01.CCM.0000201875.12245.6F 16505665

[B5] Oddo M Schmidt JM Carrera E Badjatia N Connolly ES Presciutti M Ostapkovich ND Levine JM Le Roux P Mayer SA Impact of tight glycemic control on cerebral glucose metabolism after severe brain injury: a microdialysis study Crit Care Med 2008 36 3233 3238 10.1097/CCM.0b013e31818f4026 18936695

[B6] Van den Berghe G Wilmer A Hermans G Meersseman W Wouters P Milants I Van Wijngaerden E Bobbaers H Bouillon R Intensive insulin therapy in the medical ICU N Engl J Med 2006 354 449 461 10.1056/NEJMoa052521 16452557

[B7] Van den Berghe G Wouters P Weekers F Verwaest C Bruyninckx F Schetz M Vlasselaers D Ferdinande P Lauwers P Bouillon R Intensive insulin therapy in critically ill patients N Engl J Med 2001 345 1359 1367 10.1056/NEJMoa011300 11794168

[B8] Finfer S Chittock D Su S Blair D Foster D Dhingra V Bellomo R Cook D Dodek P Henderson W Intensive versus conventional glucose control in critically ill patients N Engl J Med 2009 360 1283 1297 19318384 10.1056/NEJMoa0810625

[B9] Kinoshita K Moriya T Utagawa A Sakurai A Mukoyama T Furukawa M Yamaguchi J Tanjoh K Change in brain glucose after enteral nutrition in subarachnoid hemorrhage J Surg Res 2010 162 221 224 10.1016/j.jss.2009.06.009 19815233

[B10] Wartenberg KE Schmidt JM Claassen J Temes RE Frontera JA Ostapkovich N Parra A Mayer SA Impact of medical complications on outcome after subarachnoid hemorrhage Crit Care Med 2006 34 617 623 16521258 10.1097/01.ccm.0000201903.46435.35

[B11] Hunt WE Hess RM Surgical risk as related to time of intervention in the repair of intracranial aneurysms J Neurosurg 1968 28 14 20 10.3171/jns.1968.28.1.0014 5635959

[B12] Teasdale G Jennett B Assessment of coma and impaired consciousness. A practical scale Lancet 1974 2 81 84 4136544 10.1016/s0140-6736(74)91639-0

[B13] Knaus WA Draper EA Wagner DP Zimmerman JE APACHE II: a severity of disease classification system Crit Care Med 1985 13 818 829 10.1097/00003246-198510000-00009 3928249

[B14] Claassen J Bernardini GL Kreiter K Bates J Du YE Copeland D Connolly ES Mayer SA Effect of cisternal and ventricular blood on risk of delayed cerebral ischemia after subarachnoid hemorrhage: the Fisher scale revisited Stroke 2001 32 2012 2020 10.1161/hs0901.095677 11546890

[B15] Hijdra A van Gijn J Nagelkerke NJ Vermeulen M van Crevel H Prediction of delayed cerebral ischemia, rebleeding, and outcome after aneurysmal subarachnoid hemorrhage Stroke 1988 19 1250 1256 10.1161/01.STR.19.10.1250 3176085

[B16] Brouwers PJ Dippel DW Vermeulen M Lindsay KW Hasan D van Gijn J Amount of blood on computed tomography as an independent predictor after aneurysm rupture Stroke 1993 24 809 814 10.1161/01.STR.24.6.809 8506552

[B17] van Gijn J Hijdra A Wijdicks EF Vermeulen M van Crevel H Acute hydrocephalus after aneurysmal subarachnoid hemorrhage J Neurosurg 1985 63 355 362 10.3171/jns.1985.63.3.0355 4020461

[B18] Claassen J Carhuapoma JR Kreiter KT Du EY Connolly ES Mayer SA Global cerebral edema after subarachnoid hemorrhage: frequency, predictors, and impact on outcome Stroke 2002 33 1225 1232 10.1161/01.STR.0000015624.29071.1F 11988595

[B19] Bederson JB Connolly ES Batjer HH Dacey RG Dion JE Diringer MN Duldner JE Harbaugh RE Patel AB Rosenwasser RH Guidelines for the management of aneurysmal subarachnoid hemorrhage Stroke 2009 40 994 1025 10.1161/STROKEAHA.108.191395 19164800

[B20] Cerra FB Benitez MR Blackburn GL Irwin RS Jeejeebhoy K Katz DP Pingleton SK Pomposelli J Rombeau JL Shronts E Applied nutrition in ICU patients. A consensus statement of the American College of Chest Physicians Chest 1997 111 769 778 10.1378/chest.111.3.769 9118718

[B21] Zeger SL Liang KY Longitudinal data analysis for discrete and continuous outcomes Biometrics 1986 42 121 130 10.2307/2531248 3719049

[B22] Cook RD Weisberg S Residuals and Influence in Regression 1992 New York: Chapman & Hall

[B23] Alberda C Gramlich L Jones N Jeejeebhoy K Day AG Dhaliwal R Heyland DK The relationship between nutritional intake and clinical outcomes in critically ill patients: results of an international multicenter observational study Intensive Care Med 2009 35 1728 1737 10.1007/s00134-009-1567-4 19572118

[B24] Badjatia N Fernandez L Schlossberg MJ Schmidt JM Claassen J Lee K Connolly ES Mayer SA Rosenbaum M Relationship between energy balance and complications after subarachnoid hemorrhage J Parenter Enteral Nutr 2010 34 64 69 10.1177/0148607109348797 19884354

[B25] Zetterling M Hillered L Enblad P Karlsson T Ronne-Engström E Relation between brain interstitial and systemic glucose concentrations after subarachnoid hemorrhage J Neurosurg 2011 115 66 74 10.3171/2011.3.JNS10899 21476811

[B26] Nelson DW Thornquist B MacCallum RM Nystrom H Holst A Rudehill A Wanecek M Bellander BM Weitzberg E Analyses of cerebral microdialysis in patients with traumatic brain injury: relations to intracranial pressure, cerebral perfusion pressure and catheter placement BMC Med 2011 9 21 10.1186/1741-7015-9-21 21366904 PMC3056807

[B27] Vespa P Bergsneider M Hattori N Wu HM Huang SC Martin NA Glenn TC McArthur DL Hovda DA Metabolic crisis without brain ischemia is common after traumatic brain injury: a combined microdialysis and positron emission tomography study J Cereb Blood Flow Metab 2005 25 763 774 10.1038/sj.jcbfm.9600073 15716852 PMC4347944

[B28] Hertz MM Paulson OB Barry DI Christiansen JS Svendsen PA Insulin increases glucose transfer across the blood-brain barrier in man J Clin Invest 1981 67 597 604 10.1172/JCI110073 7009645 PMC370607

[B29] Hamai M Minokoshi Y Shimazu T L-Glutamate and insulin enhance glycogen synthesis in cultured astrocytes from the rat brain through different intracellular mechanisms J Neurochem 1999 73 400 407 10386993 10.1046/j.1471-4159.1999.0730400.x

[B30] Clarke DW Boyd FT Jr Kappy MS Raizada MK Insulin stimulates macromolecular synthesis in cultured glial cells from rat brain Am J Physiol 1985 249 C484 489 2415002 10.1152/ajpcell.1985.249.5.C484

[B31] Nakamura H Shitara N Takakura K Insulin binds to specific receptors and stimulates macromolecular synthesis in C6 glioma cells Acta Neurochir (Wien) 1988 93 10 12 10.1007/BF01409895 3046234

[B32] Hertz L Dienel GA Energy metabolism in the brain Int Rev Neurobiol 2002 51 1 102 IN101-IN104 12420357 10.1016/s0074-7742(02)51003-5

[B33] Phillis JW O'Regan MH Energy utilization in the ischemic/reperfused brain Int Rev Neurobiol 2002 51 377 414 12420365 10.1016/s0074-7742(02)51011-4

[B34] Baquer NZ Hothersall JS McLean P Function and regulation of the pentose phosphate pathway in brain Curr Top Cell Regul 1988 29 265 289 3293926

[B35] Schlenk F Graetz D Nagel A Schmidt M Sarrafzadeh AS Insulin-related decrease in cerebral glucose despite normoglycemia in aneurysmal subarachnoid hemorrhage Crit Care 2008 12 R9 10.1186/cc6776 18218076 PMC2374587

[B36] Ståhl N Ungerstedt U Nordström CH Brain energy metabolism during controlled reduction of cerebral perfusion pressure in severe head injuries Intensive Care Med 2001 27 1215 1223 10.1007/s001340101004 11534571

[B37] Zarbock SD Steinke D Hatton J Magnuson B Smith KM Cook AM Successful enteral nutritional support in the neurocritical care unit Neurocrit Care 2008 9 210 216 10.1007/s12028-008-9120-9 18654745

[B38] Oddo M Schmidt JM Carrera E Badjatia N Connolly ES Presciutti M Ostapkovich ND Levine JM Le Roux P Mayer SA Impact of tight glycemic control on cerebral glucose metabolism after severe brain injury: a microdialysis study Crit Care Med 2008 36 3233 3238 10.1097/CCM.0b013e31818f4026 18936695

